# European neurologists need to be aware of chikungunya (CHIKV) and dengue (DENV) viruses

**DOI:** 10.1007/s10072-025-08538-4

**Published:** 2025-10-11

**Authors:** Guglielmo Lucchese, Angela Stufano

**Affiliations:** 1 Department of Experimental Medicine, College ISUFI, University of Salento, Via per Monteroni, Lecce 73100 Italy; 2 Department of Neurology, University of Greifswald, Ferdinand-Sauerbruch-Straße, Greifswald, 17475 Germany; 3 Department of Medical and Surgical Sciences, University of Foggia, Ospedali Riuniti. Plesso di Medicina, Foggia, Viale Luigi Pinto 71122 Italy

Dear Prof. Tagliavini,

Data published in July 2025 on chikungunya (CHIKV) and dengue (DENV) transmission in Italy from 2006 to 2023, position the country as a European forerunner in facing arboviral outbreaks [1]. The analysis reveals that local outbreaks are primarily influenced by the timing and location of imported cases rather than by intrinsic regional susceptibility. Nevertheless, environmental factors—including urban density, climatic suitability, and the pervasive presence of Aedes albopictus—play a significant role in facilitating subsequent transmission. The majority of outbreaks occur between mid-August and mid-November, with coastal and peri-urban areas identified as particularly high-risk zones. While vector control efforts typically contain outbreaks within 15 days of detection, delayed diagnosis—especially in CHIKV cases—has in several instances allowed for multiple generations of viral spread before containment measures were implemented. The study warns that climate change and intensified global mobility are likely to drive more frequent DENV outbreaks in Europe, highlighting the necessity for enhanced surveillance in climatically favorable regions, irrespective of prior outbreak history.

Menegale et al. stress the importance of heightened physician awareness and prompt case identification as key to improving early detection and reducing future risk [1]. In particular, neurologists should maintain a high index of suspicion, as both CHIKV and DENV are associated with a broad spectrum of neurological manifestations. In a retrospective observational study, Kulkarni et al. investigated neurological complications in DENV infection among 5,821 hospitalized patients in Western India over a six-year period [2]. Of these, 154 patients (2.64%) exhibited neurological involvement. Encephalopathy was the most frequent presentation, accounting for 31.2% of cases, and was often associated with systemic disturbances such as hepatic or renal dysfunction, hyponatremia, shock, or multiorgan failure. Syncope was reported in 27.3% of patients, while encephalitis—characterized by focal neurological deficits and supported by neuroimaging or cerebrospinal fluid (CSF) abnormalities—was documented in 15.6%. Acute symptomatic seizures occurred in 11% of cases. Less common complications included intracranial hemorrhage (4.5%), Guillain–Barré syndrome (3.2%), optic neuritis (1.9%), myositis (1.3%), and hypokalemic paralysis (1.3%), with isolated cases of ischemic stroke, posterior reversible encephalopathy syndrome (PRES), myoclonus, and brachial plexopathy (0.6% each). These findings underscore the diverse involvement of both central and peripheral nervous systems in DENV infection and emphasize the importance of early recognition for appropriate clinical management.

In a comprehensive review, Fong, Wong, and Tan categorized DENV-associated neurological manifestations into four principal groups: direct viral invasion (e.g., encephalitis), metabolic complications (e.g., encephalopathy), post-infectious immune-mediated syndromes (e.g., Guillain–Barré syndrome, acute disseminated encephalomyelitis), and other less well-defined conditions such as cerebellitis and myositis [3]. The authors stressed the diagnostic importance of distinguishing encephalitis from encephalopathy due to their overlapping clinical features but differing etiologies and treatment strategies. Employing stringent criteria—including CSF analysis and virological confirmation—they found that many previously reported cases of DENV encephalitis did not meet established diagnostic thresholds, often due to serological cross-reactivity with other flaviviruses, such as Japanese encephalitis virus. The review also highlighted the limitations of current diagnostic approaches in endemic settings and called for more rigorous neuropathological studies to substantiate evidence of neuroinvasion. Guzman and Martinez expanded on the global burden of DENV-related neurological disorders in a narrative review, noting a reported prevalence ranging from 0.5% to 20% in various studies [4]. Complications included encephalitis, myelitis, Guillain–Barré syndrome, ADEM, optic neuritis, and transverse myelitis, among others. The increasing recognition of these conditions is attributed to expanding viral transmission, climate-driven spread, and the adoption of the 2009 WHO classification, which incorporates neurological involvement under severe DENV infection. The authors emphasized the urgent need for standardized diagnostic tools, predictive models, and greater awareness of potential long-term neurological sequelae, such as chronic fatigue, cognitive impairment, and even progressive dementia, which have been documented in recent case studies. They advocate for a coordinated global response, encompassing surveillance, clinical education, and the development of vaccines and therapeutics.

A 2018 systematic review comprising 856 cases of CHIKV-associated neurological disease categorized the manifestations into central (e.g., encephalitis, myelitis) and peripheral (e.g., Guillain–Barré syndrome) nervous system involvement, alongside neuro-ocular complications [5]. Encephalopathy emerged as the most common presentation (40.5%). Vertical transmission accounted for 7% of cases, with neonatal infections frequently leading to severe outcomes. While advanced age and comorbidities were recognized as risk factors, severe neurological disease was also observed in young, otherwise healthy individuals. Diagnostic challenges—particularly differentiating CHIKV from co-circulating arboviruses like DENV and Zika—were noted. A retrospective Indonesian study on CHIKV among 244 patients with acute meningoencephalitis identified four CHIKV-positive cases confirmed via RT-PCR or IgM ELISA in CSF or serum, all presenting with neurological symptoms including altered mental status and seizures, and displaying variable outcomes [6]. The findings underscore the need to consider CHIKV in the differential diagnosis of CNS infections in endemic settings, especially during outbreaks. A meta-analysis by da Costa et al. pooled data from 19 studies involving 7,319 patients to estimate the prevalence of CHIKV-associated neurological complications (CANCs), reporting a positivity rate of 12% (95% CI: 6–19%) [7]. Myelitis and ADEM were the most frequently associated syndromes (27% each), followed by Guillain–Barré syndrome (15%), encephalitis (12%), and meningoencephalitis (7%). However, significant heterogeneity was noted, owing to differences in study design, diagnostic methodologies, and geographic representation, with Brazil accounting for a disproportionate number of cases. The authors emphasized the urgent need for robust epidemiological surveillance and mechanistic studies to enhance understanding of CHIKV neurotropism and inform both clinical practice and public health strategies.

Once considered primarily tropical diseases, both DENV and CHIKV have demonstrated increasing potential for geographic expansion, including into Europe. The continued spread of Aedes albopictus and the accelerating impact of climate change are altering the epidemiological landscape of arboviral infections [8]. Modeling by Farooq et al. suggests that under extreme climate change scenarios, the risk of DENV and CHIKV outbreaks in Europe may increase nearly fivefold by the 2060s, potentially marking a transition from sporadic introductions to endemic transmission in vulnerable EU regions. Given the extensive overlap of DENV and CHIKV neurological manifestations with other infectious and non-infectious syndromes, awareness among European physicians—particularly neurologists—is critical. These emerging arboviral threats demand proactive clinical vigilance and coordinated public health responses to mitigate their evolving impact.

Sincerely,

The authors
